# Vendozoa and selective forces on animal origin and early diversification: reply to Dufour and McIlroy (2017)

**DOI:** 10.1098/rstb.2017.0336

**Published:** 2017-12-04

**Authors:** Thomas Cavalier-Smith

**Affiliations:** Department of Zoology, University of Oxford, South Parks Road, Oxford OX1 3PS, UK

## 1. Introduction

The authors are right to emphasize that Vendozoa probably include both filter feeders on plankton and those deriving nutrition directly from the underlying substratum on which they lie or move and to argue that hard and soft surfaces would have provided habitats offering partially contrasting selective forces favouring distinct body forms. However, my paper already argued that some Vendozoa were bifacial filter-feeding fronds and others likely to have been horizontal dwellers on soft surfaces that may have fed phagocytically on substrate microrganisms by ventral non-choanocyte cells and dorsally on plankton by choanocytes. A better interpretation of *Fractofusus* than that of Dufour & McIlroy [1] might be that it was just such a dorsal collar-cell and ventral substrate feeder; if so it was a presponge, not a pre-placozoan. If Ediacaran organisms of that dual feeding mode existed, the dichotomy between plankton feeders and substrate feeders was less sharp than they imply.

Their comment raises seven issues: (i) conceptually, how does the ‘pre-placozoan grade of organization’ really differ from a presponge. (ii) Can we reliably infer from fossils the actual feeding mode and different cell types of Vendozoa? (iii) What are the phylogenetic relationships between Vendozoa, sponges and Placozoa; and the inferred phenotypes prior to each branch point? (iv) What is the relative timing of vendozoan, sponge and bilaterian origins? (v) Does substrate feeding offer a transition to the first animal as plausible as the choanoflagellate to presponge path? (vi) What selective force was crucial for the origin of the nervous system? (vii) What is required for good explanations of major evolutionary transitions?

## 2. ‘Pre-placozoan grade’ is conceptually confused

McIlroy and Dufour introduced the term ‘pre-placozoan grade’ to apply to the earliest rangeomorph fossils [2], but did not define or clearly explain it. They now say pre-placozoans ‘differ from presponges by their lack of basal pinacocytes and suspension feeding capabilities, and by a feeding mode that relies on establishing symbiosis with, or directly phagocytosing chemoautotrophic bacteria’ [1]. The hypothetical nature of bacterial prey is not helpful for defining a grade of organization—a morphological concept of organismal substructure. Thus, using it implies that an organism with identical structure (thus organizational grade) feeding not on chemotrophic but on heterotrophic or photosynthetic bacteria or on eukaryotes or osmostrophically would not be of ‘pre-placozoan grade’, which is unreasonable. Excluding prey type from this definition leaves only lack of basal pinacocytes, which does not adequately define a grade of animal organization. The authors also confusingly note that compared with presponges ‘The pre-placozoan model presents a similar body plan (simple epithelium surrounding mesohyl), but with only choanocyte-like cells, involved in feeding and water circulation’ [1]. A ‘similar body plan’ is effectively the same as having a similar grade of organization, yet if they had only ‘choanocyte-like cells' their grade of organization would be that of a multicellular choanoflagellate (if ‘choanocyte-like’ means having a microvillar collar), not an animal, and thus a simpler grade than presponges. If they had only cells with collars as they imply, their dorsal cells should have been able to feed on plankton exactly like a presponge, so asserting that they did not feed on plankton but only from their ventral surface seems contradictory, and would have lowered their feeding effectiveness and thus have been selected against. Furthermore, it is unlikely that their ventral surface would retain collars, in which case saying they had only choanocyte-like cells seems wrong. Conversely, if they are not postulated to have had collars, it was wrong to call them ‘choanocyte-like’. Thus, properties suggested for ‘pre-placozoa’ are partially contradictory and/or too loosely specified.

Earlier, the authors said the vendozoan *Dickinsonia* ‘was of placozoan grade’ with ‘mucociliary sole’ [2], but neither then nor now specify how a ‘pre-placozoan grade’ differs from a ‘placozoan grade’. As I use ‘presponge’ [3], it embraces a spectrum of increasingly complex grades of organization with two to many different cell types, ranging from the simplest with two layers (true diploblasts; epithelium only) to others with mesenchyme also and thus three tissue layers (simple triploblasts). Thus, saying it ‘consisted of two cell types' [1] oversimplifies and misrepresents my paper. I would regard the most complex presponges as having a similar grade of organization to Placozoa (albeit one of their cell types being choanocytes).

## 3. Are any Vendozoa ‘pre-placozoa’?

One cannot see cells or cell types in fossil Vendozoa, so we are unsure whether they had ciliated cells or choanocytes, but can make an informed guess. Their overall size and complexity is such that it is unlikely that they were not triploblastic with the connective tissue secreted by mesenchyme cells sandwiched between two epithelia. Placozoa are also, in this sense, triploblasts, and the blanket use of diploblast for any pre-bilaterian animal [2] is thoroughly confusing and ought to cease. If they have neither gut nor aquiferous system, they can reasonably be regarded as of comparable organizational grade to Placozoa. But, it is not reasonable to regard any of them large enough to be found as fossils as of ‘pre-placozoan’ grade if that means organizationally simpler than Placozoa. In my view, all must have had substantially more complex connective tissue organization than *Trichoplax*, so the authors' idea that they were organizationally simpler than Placozoa is implausible; that Vendozoa might have had only choanocyte-like cells is incredible. If *Fractofusus* had the tissue structure postulated earlier, it would have been a complex triploblast not a diploblast as stated [2]. Calling it a pre-placozoan (without defining that term at all) allowed the reader to think they supposed it to be an evolutionary precursor of Placozoa. Their present attempt to give ‘pre-placozoan’, a meaning makes it evident that *Fractofusus* was not a pre-placozoan, so their title [2] was doubly misleading and conceptually confused.

## 4. Phylogenetic issues

Dufour & McIlroy's hypothetical tree [1] assumes that pre-placozoa gave rise to Placozoa, coelenterates and Bilateria, but not sponges; like the text, it appears to assume that the last common ancestor of pre-placozoa and sponges (i.e. all animals) was a multicell with only one somatic cell type (presumably choanocyte-like) that secreted extracellular material, yet had already evolved oogamy. It would have been effectively a multicellular choanoflagellate with oogamy. They do not suggest how this hypothetical animal ancestor fed or what selective force might have made it evolve oogamy (extremely rare in protists; unknown in any members of kingdom Protozoa) or multicellularity and is thus explanatorily empty with respect to the origin of animals. If it fed on plankton using a collar, as it must have done if choanoflagellate and sponge collar cells are homologues, it is effectively a presponge in structure and mode of feeding, so the last precursor of all animals would have been functionally a presponge, making it incorrect and misleading to say that ‘pre-placozoa’ offer an *alternative* route to the origin of animals. At best, their pre-placozoan would be relevant to the origin of the sister clade to sponges, but no good case is made for that.

The scheme also does not specify how the pre-placozoan, which supposedly evolved later than this original collar-cell feeding animal, differs from it in structure or feeding mode, and is thus equally explanatorily empty with respect to the origin of this subclade. It also has nothing to say about the origin of Placozoa, coelenterates or bilateria. Worse still, making the animal ancestor effectively a very simple organism with only one somatic cell type totally fails to account for the sharing by sponges and their sister clade of a common system of pattern formation, e.g. the Wnt antero-posterior gradient system, Notch signalling, homeobox and other spatially controlled switch genes, or the shared PIWI germline maintenance system [3]. It is implausible to argue either that such complex systems could have evolved at a choanoflagellate grade of organization with only one somatic cell type and no germline soma distinction or that they evolved separately in sponges and other animals; therefore, their fig. 1 idea of such a simple last common ancestor is almost certainly wrong. Furthermore, sponges and cnidaria/bilateria share an internal body cavity and mouth/osculum and an adult grade of organization immensely more complex than the pre-placozoan; these complexities are arguably homologous morphologically and in their pattern-forming gradient system and germ–soma distinction. Moreover, sponges and cnidaria share a common life history with a well-differentiated ciliated larva that uses aboral/aboscular secretory cells that mediate larval settling in similar ways and in sponges express numerous post-synaptic protein homologues. Despite these fundamental homologies in body plan, pattern formation and morphogenesis of both adults and larvae of sponges and cnidaria, as well as in the likely transition mechanisms between them, Dufour & McIlroy ([1], fig. 1) assume that these complexities evolved independently twice in animal evolution. That is contrary to the comparative evidence and evolutionarily incredible.

Contrary to their assertion that pre-placozoa offer an *alternative* route to sponges for the origin of animals, their fig. 1 shows it as an *additional* route, making two almost independent origins of the basic animal body plan and biphasic life cycle. This two-origin scenario doubles the complexity of animal origins. No intermediates or causes are given of how their proposed second route could have converted a one-somatic-cell ancestor directly into a cnidarian or bilaterian as fig. 1 implies, so it is a non-starter as an ‘explanation’. Merely calling their hypothetical common ancestor a ‘pre-placozoan’ without specifying even one way it differs from its ancestor shared with sponges is not an explanation. It is also misleading to call this entity a pre-placozoan which implies that it was a direct ancestor of placozoa, when it is likely that Placozoa evolved by simplification from a much more complex ancestor with numerous cell types. It was no more sensible to call it a pre-placozoan than a pre-coelenterate or pre-bilaterian if the authors' tree is correct.

Their fig. 1 arbitrarily put ‘basal pinacocytes’ at the base of the sponge-only lineage; they did not explain why they could not have been put below the basal fork and why their pre-placozoan could not have evolved from a presponge with two somatic cell types. The authors acknowledge that colonization of the surface of soft marine sediments is challenging, but do not explain how a multicellular choanoflagellate could have overcome those challenges or tell us anything about intermediates.

Oddly given their palaeontological expertize, they did not place Vendozoa on their fig. 1 which makes it hard to fathom what they really think is the relevance of Vendozoa to either the origin of animals or the primary bifurcations of extant groups, which limits one's ability to sort the wheat from the chaff in their comment, but I assume they would have put them as sister to the placozoa/cnidaria/bilateria clade had they not preferred to hide their view by omitting them. My own interpretation was that Vendozoa are derivatives of a complex triploblastic presponge grade of organization (with connective tissue as complex as Cnidaria or sponges), but without aquiferous or nervous system or gut or nematocysts, which had acquired triploblastic tissue organization by the choanoflagellate to advanced presponge route, but diversified to fill a variety of nutritional niches before a gut or nervous system evolved. None of their references to the fossil record contradicts that view. Non-descript *Thectardis* might, as they suggest, be a sponge, but it is hard to say what it is—if it is an early animal, its size suggests triploblasty not a pre-placozoan grade. It is plausible that *Blackbrookia* is a sponge before mineralized spicules evolved, but one cannot be sure.

## 5. New palaeontological evidence on relative timing of eukaryotic kingdom origins

A recent study (taking more care to exclude modern contaminants than before) concluded that 24-isopropylcholestane (ipc) sometimes supposed to be a specific marker for demosponges does not extend backwards prior to approximately 650 Ma in the extensive interglacial between the major Sturtian and shorter Marinoan Snowball-Earth episodes in the Cryogenian period [4]. If ipc really were a specific demosponge marker, that would mean that sponges originated a few tens of millions of years before Vendozoa and ought to be widespread in the Ediacaran fossil record, yet possible sponges are sparse (likely stem not crown sponges) and convincing demosponges or hexacts absent. However, as Antcliffe emphasized [5], ipc is also made in large amounts by pelagophyte algal chromists, a deep branching ochrophyte lineage, so its sudden late-Cryogenian rise might be attributable to the origin of planktonic ochrophyte algae, not sponges. That is quite plausible as ipc first appeared abundantly at precisely the same time as stigmastane, held to mark the origin of Viridiplantae, which are sisters of red algae whose symbiogenetic enslavement originated kingdom Chromista (oddly not mentioned by Brocks *et al.* [4]), the sister of kingdom Plantae, which most likely happened rapidly after the origin of red algae—necessarily close to the origin of Viridiplantae [6,7]. Thus, evidence from ipc is asymmetric: its absence before 650 Ma makes it unlikely that sponges or other animals evolved before then, but its appearance then does not require sponges to be that old or older than or as old as Vendozoa. Probably, the simplest presponges were slightly older than Vendozoa.

This conclusion is unaffected by an unconvincing claim that pelagophytes evolved the ability to make ipc 100 Myr after demosponges [8]. That was based on assuming that this now unquestioned independent ipc origin required independent gene duplications for carbon-24/28 sterol methyltransferase (SMT), for which there is no direct evidence as the exact enzymes used and their degree of multifunctionality (widespread in steroid synthesis) are unknown, combined with a flawed attempt to date sponge and chromist SMT duplications [8]. Motivating logic was reasonable, but the conclusion was entirely invalid as methods were extremely biased: their protein-sequence tree (fig. S3A) grouped the third pelagophyte paralogue with 79% support not with other heterokont (=stramenopile, a regrettable junior synonym) sequences, but with that of the alveolate *Perkinsus*, showing it is not a recent intra-heterokont duplication, but probably occurred before the last common ancestor of Halvaria (heterokonts plus alveolates). Yet, their fossil-calibrated molecular ‘clock’ analysis did not use this protein tree, but a taxonomically much sparser one from which *Perkinsus* and all other non-heterokont chromist sequences (originally too sparsely sampled) were omitted and used less reliable nucleotide not amino acid sequences. On that unwisely culled tree, the pelagophyte third paralogue had no close relative, so switched its position from being with heterokont clade 2 to beside heterokont clade 1 (fig. S3B). Worse still, the authors did not even use that tree for their analysis, but first put it through a programme (NOTUNG) that changed its branching order (!) to minimize duplications that moved the pelagophyte paralogue 3 into heteterokont paralogue 1 as sister to pelagophyte paralogue 2, making it falsely appear in fig. 3 to result from a relatively recent duplication, the exact opposite of what the more reliable, unmanipulated protein tree (fig. S3A) shows. Even if the input had not been thus topologically seriously distorted, using any single-gene paralogue tree to date events would likely have been highly unreliable (the grossly inflated earlier dates on their fig. 3 are not credible, such backward extrapolation being far too model-dependent to be useful); by comparison with multigene trees, their heterokont branching order was completely wrong for both paralogues.

If the third halvarian paralogue really does add the third methyl to make pelagophyte ipc [8] (questionable), its origin would be better dated by mapping it onto a multiprotein tree (e.g. [7], fig. 2); from that tree for 187 proteins and 171 eukaryotes (opisthokonts and chromists much more richly sampled than in [8]), Halvaria appear substantially older than opisthokonts, which (*if* the authors' assumption of the significance of that duplication is correct) makes it likely that the oldest ipc signal was not from sponges, but from halvarian algae and demosponges evolved significantly after 650 Ma. Thus, new sterane data [4] are compatible with my suggestion that presponges and thus animals originated at the end of the Cryogenian [3], likely almost immediately after Marinoan melting removed the last inhibitory ice-house conditions that previously likely restrained diversification of pre-existing protozoan phylum Choanozoa; I suggest that choanoflagellates (not the oldest Choanozoa) arose then, before which animal origin was evolutionarily impossible, because the transition was too hard selectively through any route other than choanoflagellates to sponges.

It may also have been impossible before then for environmental reasons, because the latest biogeochemical theory of atmospheric oxygen levels argues for three separate long-enduring metastable global steady states differing by over two orders of magnitude (Archaean low *p*O_2_; most Proterozoic intermediate *p*O_2_; Ediacaran/Phanerozoic high *p*O_2_) separated by two sudden Snowball-Earth destabilizing eras that rapidly increased oxygen levels; and that Cryogenian snowball perturbations switched the medium level to the present highest level [9]. Though that inferred sudden rise in oxygen presumably provided a permissive environment for animal evolution for the first time [9], I argue that the concomitant origin of choanoflagellates and their unique filter-feeding was the key positive stimulus by providing the only suitable cellular precursors.

This scenario is not affected by the fact that a novel sterane, tentatively identified as 26-methylcholestane (cryostane) and suggested as a demosponge marker [10], is restricted to the later part of the immediately preCryogenian Tonian period. Demosponges being the only organisms known to methylate sterols in the 26-position is not convincing evidence that they made that cryostane, especially as sponges thus methylate ingested ergostane and stigmastane (absent before 650 Ma) not cholestane, and thus do not synthesize the right steroid precursor for making cryostane. If despite those objections cryostane were from demosponges, why should it have disappeared from the fossil record approximately 720 Ma? As no analyses exist for sterols in most heterotrophic protist lineages, some might make cryostane. More likely, cryostane was made by a now extinct protozoan that flourished only approximately 770–720 Ma, e.g. the marine vase-shaped probable testate amoebae like *Melanocyrillium* that I argued were probably an extinct early group of amoebae [11], not arcellinids (as palaeontologists assumed) which are exclusively freshwater. These flask-shaped tests closely fit cryostane's temporal duration [12].

If the sudden jump in sterane/hopane ratios at 650 Ma and simultaneous onset of the ipc record in the Sturtian/Marinoan interglacial represents the origin of Plantae [4] and Chromista, then the simultaneous origin of 24-*n*-propylcholestane, a putative marker for the heterotrophic chromist infrakingdom Rhizaria [4], is precisely what is expected from the chromist diversification pattern on multigene trees [7]. That makes it likely that neokaryotes (the clade comprising animals, fungi, protozoan subkingdom Neozoa, Plantae and Chromista [6]) substantially diversified in a neokaryote explosion approximately 650 Ma. The absence of fungi and choanoflagellates before then, both able to make ergosterol, would account for ergostane rarity. Before neokaryotes originated, eukaryotes probably comprised only heterotrophs from protozoan subkingdom Eozoa (phyla Euglenozoa, Percolozoa and Eolouka [6]), of which kinetoplastid Euglenozoa at least make ergosterol and could have contributed to the isolated late Tonian ergostane occurrences (though if contrary to my assumption, the root of the eukaryote tree is not within Eozoa, but between Eozoa and neokaryotes (as one sequence tree suggested), then the pre-650 Ma sterane record dominated by cholestanes might have come largely or entirely from stem eukaryotes not Eozoa). If neokaryotes originated just after or just before the Sturtian glaciation, the somewhat earlier vase-shaped fossils would have represented an extinct eozoan group not early Amoebozoa.

The now decontaminated sterane fossil record [4], including the conspicuous absence of steranes (thus eukaryotes) from an approximately 820 Ma hypersaline habitat where the oldest convincing isoprenoid evidence for archaebacteria (in my view, sisters not ancestors or eukaryotes) was found [13], fits my longstanding conclusion that crown eukaryotes and archaebacteria are both substantially younger than many palaeontologists think and that all fossils older than approximately 810 Myr identified as crown eukaryotes, e.g. *Bangiomorpha* claimed to be a red alga, were misidentified [12,14]. However, sterane data before 820 Ma are still absent, but essential to test this more rigorously (and likely disprove the entirely unfounded dogma of archaebacterial antiquity). Even the present evidence shows that eukaryotes had only a minor ecological role before 650 Ma and a much narrower range of steranes than modern eukaryotes, all simpler C_26_, C_27_ cholestanes except for cryostane unknown from crown eukaryotes, as one might expect if they were largely stem eukaryotes.

As Mills & Canfield plausibly explain [15], the origin of efficient planktonic filter-feeding by sponges (and I now suggest by the more complex presponge frondose Vendozoa) probably decreased the availability of their picoplankton prey and simultaneously seeded shallow sediments with novel detrital microparticles that would have increased the food supply and selective advantage for benthic microbes and sediment-feeding animals, an ecosystem engineering event amplified by the almost immediate consequential origin of coelenterates (enabled by the complex body plan and pattern formation of their putative stem sponge ancestors), thereby magnifying the adaptive zone available for through-gut vermiform bilateria, which likely evolved soon afterwards and diversified rapidly in the late Ediacaran, generating the early Cambrian fossil explosion—a virtually inevitable tertiary consequence of triploblasty, neurogenesis and the gut, not the direct result of multicellularity *per se*. Thus, for these ecological reasons, as well as on the tissue evolution principles I emphasized [3], it makes most sense for benthic feeders to have followed sponge-like planktonic filter feeders.

## 6. Huge feeding capacity loss in the pre-placozoan model

Contrary to the unjustified assertion that the pre-placozoan ‘actually shows no reduction in feeding capacity’ at the unicellular to multicellular transition [1], it implies a halving of feeding capacity, as they assume that its dorsal epithelial cells do not engage in feeding by collar cells and only the ventral epithelium feeds on substrate microbes. Compared with a multicellular choanoflagellate ancestor where all cells ingest, it would be at a twofold selective disadvantage and thus could not have evolved without a compensating selective advantage. As the authors failed to recognize their model's inherent twofold disadvantage, they did not even try to propose a compensating advantage or indeed specify any selective advantage of muticellularity. As I stressed, though evolving multicellularity is mechanistically easy (cell surface glue), it is normally selected against, which is why there are hundreds of thousands of unicell species, and can only evolve with a substantial selective advantage [3]; their scenario beautifully exemplifies the scores of disparate ideas put forward for animal origins that avoid specifying the selective advantage that favoured the assumed intermediate state, which usually would be disadvantageous making them causally non-explanations of the problem. The choanoflagellate to sponge pathway remains the only one that allows a transition to animal multicellularity with several somatic cell types without gross selective disadvantage.

Dufour & McIlroy [2] did not originally argue that a *Fractofusus*-like animal was the first animal. Their new idea that a pre-placozoan could have originated directly from a multicellular choanoflagellate and gave rise directly to cnidaria is evolutionarily unsatisfactory through omitting detailed intermediate stages and selective advantage arguments. The proposed feeding modes (intracellular symbiogenesis of chemosynthetic bacteria or bacterial phagotrophy on soft sediments) are extensively exploited by hordes of different protists, but no multicells lie or move on surfaces feeding in the proposed manner. In my view, it is more efficient to do both as a unicell and selection would act predominantly *against* evolving such multicelluarity and thus prevent, not favour, it; they mention multicellularity increasing surface area, but it actually reduces the surface area to volume ratio compared with unicellularity. No argument was given why multicellularity mutants relying nutritionally on ventral epithelial feeding could survive by abandoning dorsal collar-based filter-feeding in competition with competitors that retained it in addition to ventral feeding, as their new model implicitly assumes.

However, after the difficult transition to triploblasty was *already* made by the presponge route on hard substrates, the situation would be entirely different. *Then*, flat quilted *Fractofusus*-like organisms could have colonized previously unexploited soft muddy substrates by filter-feeding triploblastic precursors detaching from solid surfaces, flopping down onto the mud and focusing ventrally on epithelial non-filter-feeding phagotrophy (like *Trichoplax*) as I already proposed [3] and maybe also getting nutrients from intracellular symbionts for the reasons emphasized by Dufour & McIlroy [2]. That would exploit a new adaptive zone through a highly developed precursor with far greater chance of success than a simple pre-placozoan; this habitat change would have evaded competition from their vertical bifacially choanocyte-feeding forbears, so they could have evolved even if losing collars ventrally initially lowered feeding efficiency. But, it would be better for such benthic feeders to have retained dorsal collar-based feeding to minimze the cost in lost food, so I do not understand why Dufour and McIlroy insist on total loss of choanocytes—to make them appear not to have been of advanced presponge grade, as I considered them?

## 7. Neurogenesis and selective forces

Seemingly to undermine my explanation of neurogenesis, McIlroy and Dufour asserted that ‘the mode of life of the presponges and sponges as suspensivores/osmotrophs on hard surfaces is simple and has few selection pressures requiring the evolution of a nervous system’ [1], which embodies several evolutionary fallacies. First is the centuries-old myth of sponge simplicity compared with other animals, which valiant efforts of sponge specialists for decades (e.g. [16–18]) have evidently not yet expunged from the secondary zoological literature, as it should be. Second is the quasi-Lamarckian misconception that selection ‘requires’ evolution of complex characters. Mutations cause change; selection is simply the metaphor for the inevitable dying out of those reducing reproductive success and multiplication of those whose effects on development increase organismal reproductive success, not a separate force of nature [19]. It does not matter that there may be only ‘few’ selective forces favouring something. Only one reason is needed to favour a novelty decisively when it arises. The authors ignore my suggestion that the decisive step in nervous system origins was modifying the settling mechanism of sponge larvae, converting their flask cells into nematocytes, initially for improving a pre-existing feature of the complex life cycle: better coordination between larval sensors detecting a good place to settle and secretion of extracellular glue to fix the adult [3]. Sensory control over settling, when sensors and effectors are separate cells, is a shared feature of the almost equally complex biphasic life cycle of sponges and Cnidaria, which would not have been shared by the purely benthic ‘pre-placozoan’. It was probably later recruitment of incipient nervous control of proto-nematocytes for a novel feeding mode on larger prey that perfected the nervous system [3].

Even if later in history the same set of early mutations occurred in other sponges, they would not have evolved nematocyst-mediated carnivory and a true nervous system, because crude beginning forms like those arguably successful in the absence of Cnidaria could not have competed with fully evolved Cnidaria, so selection would have prevented repetition of earlier events (a major principle in evolution: the chief spoils go to whoever first collars the market and excludes start-up competitors; the harder it is to duplicate the key innovation or steal it by symbiogenesis as first plants and then chromists did for chloroplasts [6] the more likely is enduring monopoly, best exemplified by the single origin of eukaryotes, the hardest step in all evolution [20]). Therefore, when cladorhizid demosponges independently evolved carnivory, they evolved neither nematocysts nor a true nervous system, but some were so radically changed as to lose the aquiferous system [21], yet unlike stem cnidarians did not originate a suite of new phyla as all major animal adaptive zones were already filled. That proves that sponges have the potential to become carnivores and radically change their body plan, but consequences of such changes inevitably differ depending on what other organisms are present. (Incidentally, glass sponges evolved action potentials independently of Cnidaria and some glass sponges colonized soft surfaces secondarily.) The authors neither argue against my neurogenesis idea nor suggest why or how a far simpler pre-placozoan instead might evolve a nervous system or offer any reason why it should have had a complete post-synaptic scaffold, as was present in the last common ancestor of sponges and other animals [22].

## 8. Requirements for good explanations of major evolutionary transitions

I have explained why the comment altogether fails to explain the origin of animals or any major extant subgroups or to make a case for the feeding mode postulated for *Fractofusus* being that of the last common ancestor of the placozoa/Cnidaria/Bilateria clade; and why their assumed diphyletic origin of animals is almost certainly wrong and the pre-placozoan idea imprecise and confused. Yet, on the positive side, their idea of how *Fractofusus* fed might be partially correct, and I agree that both substrate phagotrophy and endosymbiosis of chemosynthetic bacteria are possible nutritional modes for some Vendozoa. But, one must not confuse nutritional mode with body plan or organizational grade or confuse the diversification of Vendozoa with either the origin of animals or the causes of the Cambrian explosion: three distinct questions. It is also essential to consider selective advantages of proposed major transitions; to specify assumed intermediate stages in more explicit detail than they did; to recognize molecular and developmental evidence for animal organizational unity; and to have more respect for Occam's razor, which can readily cut away most of what has been written on animal origins. Evolutionary hypotheses should be explicit and detailed to facilitate reasoned criticism, refutation and improvement; not non-committal and vague to evade referee objections which can make them untestable and scientifically useless.

## References

[1] Dufour SC, McIlroy D 2017 An Ediacaran pre-placozoan alternative to the pre-sponge route towards the Cambrian explosion of animal life: a comment on Cavalier-Smith 2017. Phil. Trans. R. Soc. B 373, 20170148 (10.1098/rstb.2017.0148) PMC5717534 29203719

[2] Dufour SC, McIlroy D 2017 Ediacaran pre-placozoan diploblasts in the Avalonian biota: the role of chemosynthesis in the evolution of early animal life. In Earth system evolution and early life: a celebration of the work of Martin Brasier (eds Brasier AT, McIlroy D, McLoughlin N), vol. 448. London, UK: Geological Society of London.

[3] Cavalier-Smith T 2017 Origin of animal multicellularity: precursors, causes, consequences—the choanoflagellate/sponge transition, neurogenesis and the Cambrian explosion. Phil. Trans. R. Soc. B 372, 20150476 (10.1098/rstb.2015.0476) 27994119 PMC5182410

[4] Brocks JJ, Jarrett AJM, Sirantoine E, Hallmann C, Hoshino Y, Liyanage T 2017 The rise of algae in Cryogenian oceans and the emergence of animals. Nature 548, 578–581. (10.1038/nature23457) 28813409

[5] Antcliffe J 2013 Questioning the evidence of organic compounds called sponge biomarkers. Palaeontology 56, 917–925. (10.1111/pala.12030)

[6] Cavalier-Smith T In press. Kingdom Chromista and its eight phyla: a new synthesis emphasising periplastid protein-targeting, cytoskeletal and periplastid evolution, and ancient divergences. Protoplasma. (10.1007/s00709-017-1147-3) PMC5756292 28875267

[7] Cavalier-Smith T, Chao EE, Lewis R 2015 Multiple origins of Heliozoa from flagellate ancestors: new cryptist subphylum Corbihelia, superclass Corbistoma, and monophyly of Haptista, Cryptista, Hacrobia and Chromista. Mol. Phylogenet. Evol. 93, 331–362. (10.1016/j.ympev.2015.07.004) 26234272

[8] Gold DA, Grabenstatter J, de Mendoza A, Riesgo A, Ruiz-Trillo I, Summons RE 2016 Sterol and genomic analyses validate the sponge biomarker hypothesis. Proc. Natl Acad. Sci. USA 113, 2684–2689. (10.1073/pnas.1512614113) 26903629 PMC4790988

[9] Laakso TA, Schrag DP 2017 A theory of atmospheric oxygen. Geobiology 15, 366–384. (10.1111/gbi.12230) 28378894

[10] Brocks JJ, Jarrett AJ, Sirantoine E, Kenig F, Moczydlowska M, Porter S, Hope J 2016 Early sponges and toxic protists: possible sources of cryostane, an age diagnostic biomarker antedating Sturtian Snowball Earth. Geobiology 14, 129–149. (10.1111/gbi.12165) 26507690

[11] Porter SM, Meisterfeld R, Knoll AH 2003 Vase-shaped microfossils from the Neoproterozoic Chuar group, Grand Canyon: a classification guided by modern testate amoebae. J. Paleont. 77, 409–429. (10.1017/S0022336000044140)

[12] Cavalier-Smith T 2013 Early evolution of eukaryote feeding modes, cell structural diversity, and classification of the protozoan phyla Loukozoa, Sulcozoa, and Choanozoa. Eur. J. Protistol. 49, 115–178. (10.1016/j.ejop.2012.06.001) 23085100

[13] Schinteie R, Brocks JJ 2017 Paleoecology of Neoproterozoic hypersaline environments: biomarker evidence for haloarchaea, methanogens, and cyanobacteria. Geobiology 15, 641–663. (10.1111/gbi.12245) 28691279

[14] Cavalier-Smith T 2006 Cell evolution and earth history: stasis and revolution. Phil. Trans. R. Soc. B 361, 969–1006. (10.1098/rstb.2006.1842) 16754610 PMC1578732

[15] Mills DB, Canfield DE 2017 A trophic framework for animal origins. Geobiology 15, 197–210. (10.1111/gbi.12216) 27686422

[16] Leys S 2015 Elements of a ‘nervous system’ in sponges. J. Exp. Biol. 218, 581–591. (10.1242/jeb.110817) 25696821

[17] Leys SP, Hill A 2012 The physiology and molecular biology of sponge tissues. Adv. Mar. Biol. 62, 1–56. (10.1016/B978-0-12-394283-8.00001-1) 22664120

[18] Leys SP, Nichols SA, Adams ED 2009 Epithelia and integration in sponges. Integr. Comp. Biol. 49, 167–177. (10.1093/icb/icp038) 21669855

[19] Cavalier-Smith T 2010 Mutationism and the reproductive struggle: a fresh evolutionary synthesis beyond Neodarwinism In Darwin's Heritage Today: Proc. of the Darwin 200 Beijing Int. Conf., pp. 44–59. Beijing: Higher Education Press.

[20] Cavalier-Smith T 2014 The neomuran revolution and phagotrophic origin of eukaryotes and cilia in the light of intracellular coevolution and a revised tree of life. In The origin and evolution of eukaryotes (eds Keeling PJ, Koonin EV), pp. 41–77. Cold Spring Harbor, NY: Cold Spring Harbor Laboratory Press. 10.1101/cshperspect.a016006 PMC4142966 25183828

[21] Riesgo A, Taylor C, Leys SP 2007 Reproduction in a carnivorous sponge: the significance of the absence of an aquiferous system to the sponge body plan. Evol. Dev. 9, 618–631. (10.1111/j.1525-142X.2007.00200.x) 17976057

[22] Sakarya O, Armstrong KA, Adamska M, Adamski M, Wang IF, Tidor B, Degnan BM, Oakley TH, Kosik KS 2007 A post-synaptic scaffold at the origin of the animal kingdom. PLoS ONE 2, e506 (10.1371/journal.pone.0000506) 17551586 PMC1876816

